# Case Report: Case studies evaluating anti-HER2 therapy as a tumor-agnostic strategy in rare malignancies

**DOI:** 10.3389/fonc.2026.1745419

**Published:** 2026-04-30

**Authors:** Piyanoot Jitthiang, Rattanavit Phandthong, Ployploen Phikulsod, Siwanon Jirawatnotai, Narongsak Kiatikajornthada, Harit Suwanrusme

**Affiliations:** 1 Horizon Cancer Center, Bumrungrad International Hospital, Bangkok, Thailand; 2 Faculty of Medicine, Chulalongkorn University, Bangkok, Thailand; 3 Hematology Unit, Department of Internal Medicine, Faculty of Medicine, Siriraj Hospital, Mahidol University, Bangkok, Thailand; 4 Department of Pharmacology, and Siriraj Center of Research Excellence for Precision Medicine and Systems Pharmacology, Faculty of Medicine, Siriraj Hospital, Mahidol University, Bangkok, Thailand

**Keywords:** HER2-targeted therapy, invasive poorly differentiated carcinoma, periampullary cancer, rare cancer, salivary duct carcinoma

## Abstract

Despite the success of HER2-targeted antibody-drug conjugates (ADCs) in common cancers, there is a lack of real-world evidence regarding their role in rare tumors where *ERBB2* gene amplification by next-generation sequencing (NGS) may serve as a more precise driver than traditional protein expression. In this retrospective case series at Bumrungrad International Hospital, we describe three patients with *ERBB2*-amplified rare tumors—periampullary carcinoma (immunohistochemistry [IHC] 2+, 77 copies by NGS), salivary duct carcinoma (IHC 3+, 33.7 copies), and poorly differentiated carcinoma (IHC 1+, 16.8 copies)—treated with trastuzumab emtansine (T-DM1) and/or trastuzumab deruxtecan (T-DXd). Despite heterogeneous histology and low-to-equivocal IHC scores, all achieved meaningful objective responses: a 301-day partial remission, a complete intracranial and systemic response using sequential ADC therapy (T-DXd followed by T-DM1 after toxicity), and a near-complete response in an IHC 1+ (HER2-low) tumor. These results demonstrate that high-level gene amplification can predict ADC efficacy regardless of IHC status or rare histology. Our findings underscore the value of integrating NGS into diagnostic workflows at the point of care to identify ‘hidden’ responders and support the feasibility of ADC sequencing in the management of treatment-limiting toxicities.

## Introduction

The HER2 transmembrane receptor tyrosine kinase, encoded by *ERBB2* gene, is a critical cancer target, validated across breast, gastric, and esophageal cancers. Therapeutic strategies include small-molecule inhibitors, monoclonal antibodies, and anti-body-drug conjugates (ADCs). ADCs are specialized therapeutic agents consisting of a monoclonal antibody (such as trastuzumab) chemically linked to a cytotoxic payload. These molecules are designed to deliver potent chemotherapy specifically to cells expressing the target antigen, thereby increasing the therapeutic index and reducing systemic toxicity. Recently, the HER2-directed ADC trastuzumab deruxtecan (T-DXd) received tumor-agnostic FDA approval for late-line treatment of HER2-positive (immunohistochemistry [IHC] 3+) solid tumors, regardless of tumor origin (1). T-DXd is a next-generation ADC utilizing a topoisomerase I inhibitor payload and a high drug-to-antibody ratio of approximately 8. Its unique peptide-based linker is cleavable by lysosomal enzymes, allowing the payload to cross cell membranes and exert a bystander effect on neighboring cancer cells, regardless of their individual HER2 expression levels (2). Alongside this, trastuzumab emtansine (T-DM1)—an earlier generation ADC consisting of trastuzumab linked to the microtubule inhibitor DM1 (maytansinoid)—has demonstrated efficacy in breast cancer, and limited efficacy in gastric and non-small cell lung cancer (3, 4).

Interestingly, T-DXd is the first HER2-targeted therapy to show significant clinical benefit in HER2-low disease (defined as an IHC score of 1+, or 2+ with negative *in situ* hybridization [ISH]) in metastatic breast cancer (5). More recently, the DESTINY-Breast06 trial (6) explored the efficacy of T-DXd in HER2-ultralow disease. This category is defined as IHC 0 with faint, incomplete membrane staining in ≤10% of tumor cells, distinguishing it from the IHC 0 with no staining category (6). While the current FDA tumor-agnostic approval for T-DXd is indicated for patients with HER2-positive (IHC 3+ or IHC 2+/ISH+) malignancies, these data suggest that the clinical benefit of HER2-targeted ADCs may extend to a continuum of low-level expression previously categorized as HER2-negative.

Moreover, they underscore the need for refined HER2 testing strategies to accurately identify eligible patients and prompt critical discussion on the optimal methodologies for assessing HER2 expression. While IHC and ISH remain the gold-standard techniques for evaluating HER2 protein overexpression and gene amplification, next-generation sequencing (NGS) and polymerase chain reaction (PCR) offer valuable complementary approaches—particularly in the context of *ERBB2* (the gene encoding for HER2 protein)-mutated or amplified tumors.

Patients with rare cancers often face limited treatment options, largely due to an incomplete understanding of their underlying biology. These malignancies frequently harbor distinct molecular alterations that remain poorly characterized, and the scarcity of tumor samples and clinical data further hinders the development of effective, evidence-based therapies. The rapid evolution of HER2-targeted treatment strategies introduces a promising new paradigm. This therapeutic advancement has the potential to extend clinical benefit to subsets of rare cancers with HER2 expression.

We present a retrospective case series of three rare malignancies—periampullary cancer, salivary duct carcinoma, and invasive poorly differentiated carcinoma—at Bumrungrad International Hospital that exhibited favorable responses to HER2-targeted therapies. Treatment outcomes are correlated with HER2 expression profiles, highlighting the potential therapeutic relevance of HER2-directed strategies in these rare tumor types.

Response was assessed using Response Evaluation Criteria in Solid Tumors (RECIST 1.1), defining overall response rate (ORR; comprising complete response [CR] and partial response [PR]), stable disease (SD), and progressive disease (PD). Outcomes included progression-free survival (PFS), overall survival (OS), and Time on Treatment. Adverse events were scored via Common Terminology Criteria for Adverse Events (CTCAE) v5.0.

## Case presentations

Case 1 A 70-year-old female was diagnosed with stage I clear cell endometrial carcinoma and stage I periampullary cancer simultaneously. She underwent total hysterectomy, bilateral salpingo-oophorectomy, and pancreaticoduodenectomy (Whipple procedure), followed by vaginal cuff closure. The patient achieved a durable clinical complete remission lasting four years, until surveillance imaging revealed an oligometastatic disease to the liver by computed tomography (CT) scan. Biopsy confirmed recurrent periampullary adenocarcinoma. IHC profiling demonstrated a CK7+/CK20+ immunophenotype with CDX2 negativity. The patient decided to receive proton beam radiotherapy to hepatic metastases without systemic treatment.

The disease remained in clinical remission for six months. Subsequently, an elevated tumor marker level was noted, with CA19–9 measured at 194.9 U/mL. Positron Emission Tomography–Computed Tomography (PET-CT) demonstrated disease progression to the lungs and liver. A core needle biopsy of the left lower lung nodule was performed, and histopathological examination confirmed metastatic involvement of the lung. HER2 IHC analysis (clone 4B5, Ventana) yielded an equivocal score of 2 +. The tumor demonstrated moderate-to-intense membranous and cytoplasmic staining in >90% of viable cells, based on the 2018 ASCO/CAP breast cancer criteria (7). IHC slides were unavailable due to external diagnostic processing prior to referral. Due to limited tissue availability, ISH was not performed. Tissue-based NGS via the FoundationOne CDx comprehensive genomic profiling (CGP) assay identified a high-level *ERBB2* amplification (68 copies) without detectable mutations. Additionally, amplifications in *CCNE1, PIK3C2B*, and *MDM4* were noted, along with a *TP53* R273C point mutation.

**Figure 1:**
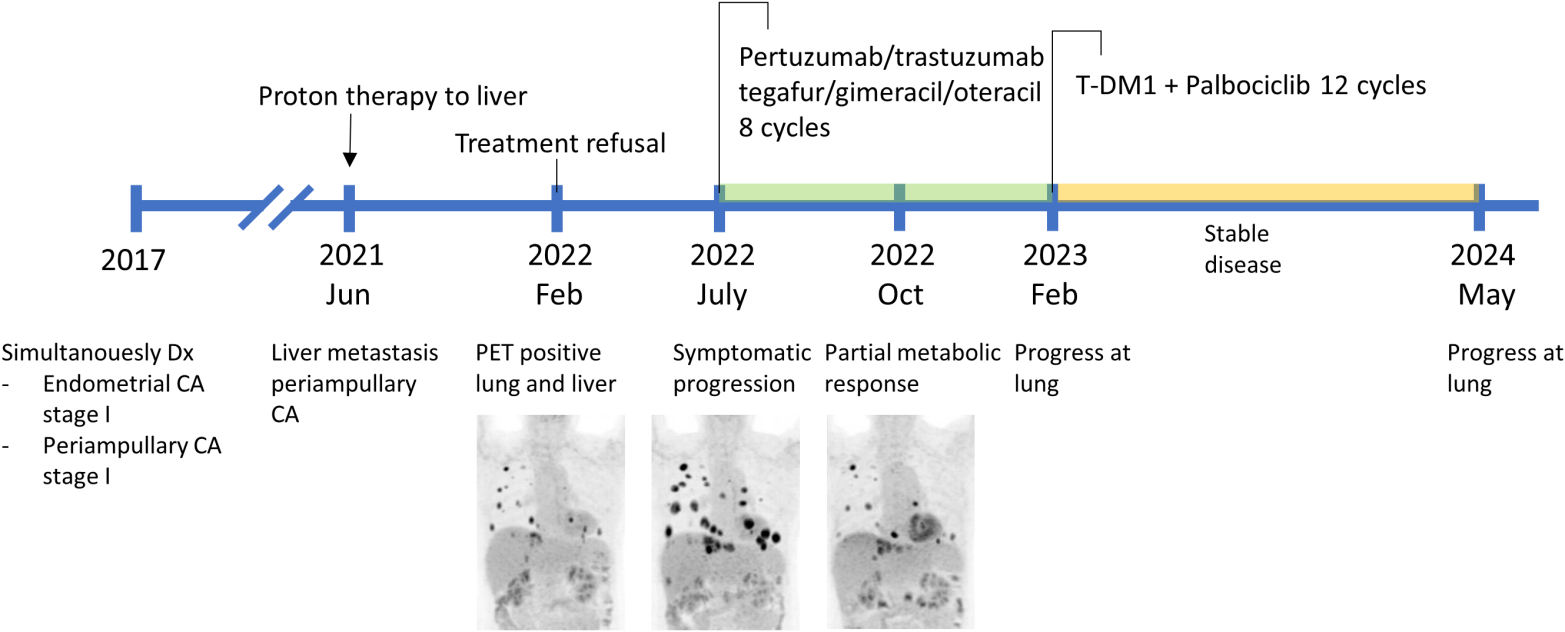
Timeline of diagnosis and treatment for case 1.

Prior to the availability of T-DXd in the country, due to HER2 positivity, Pertuzumab/trastuzumab (Phesgo) in combination with tegafur/gimeracil/oteracil (TS-1) for eight cycles were given. The patient experienced tolerable toxicity, limited to grade 1 oral mucositis, and subsequently achieved a partial remission. Upon disease progression, characterized by an increase in both the size and number of lung metastases, liquid biopsy with NGS (FoundationOne Liquid CDx) revealed amplifications in *ERBB2* (77 copies with no detected mutation)*, MDM2, PIK3C2B*, and *CCNE1*, as well as a *TP53* R273C point mutation. A combination of T-DM1 and palbociclib was then initiated every three weeks for 12 cycles, achieving a stable disease for 14 months (**Figure 1**). Throughout this period, she maintained a good quality of life with no treatment-related toxicities exceeding grade 2. At the time of progression, T-DXd was recommended. Unfortunately, her performance status declined and was further complicated by infectious events, prompted a transition to palliative care. A summary of clinical characteristics and treatment outcomes in the patients is listed in **Table 1**.

**Table 1:** Clinical characteristics, molecular profiles, and treatment outcomes of patients receiving HER2-targeted therapies for HER2-amplified advanced malignancies.

Diagnosis	Age	Stage	Tissue	HER2 Expression		Additional genetic mutation	Status	Previous treatment	Anti-HER2	Outcome	Duration of response
	Sex			IHC	NGS						
Periampullary CA	70 F	IV	Liver biopsy 7/7/21	2+	*ERBB2* amplification by tissue biopsy NGS (68 copies), and liquid biopsy NGS (77 copies)	*CCNE1* amplification, *MDM4* amplification(equivocal), *PIK3C2B* amplification(equivocal), *TP53* R273C	R/R	Phesgo (trastuzumab and Pertuzuamab) +TS-1	T-DM1	partial response	301 days
Salivary duct carcinoma	65 M	IV	Jugular lymph node biopsy 16/5/24	3+	*ERBB2* amplification by tissue biopsy NGS (33.7 copies)	*TP53* K132N, *BRCA1* heterozygous deletion	R/R	paclitazel/carboplatin/trastuzumab for 4 cycles then pembrolizumab and trastuzumab for 11 cycles	T-DXd	complete response	141 days
Poorly differentiate carcinoma	28 M	IV	Soft tissue(back) biopsy on 23/11/23	1+	*ERBB2* amplification by liquid biopsy NGS (16.8 copies)	*MYC* A59M, *TP53* R248Q, *PIK3CA* H1047R*, FGFR1* T657I, *ERBB2* V1085, *ERBB2* E695*, *ERBB2* D769H, *ERBB2* R677Q, *CDKN2A* copy number loss, *CDKN2B* copy number loss	1^st^ line		T-DXd	partial response	42days

**Figure 2:**
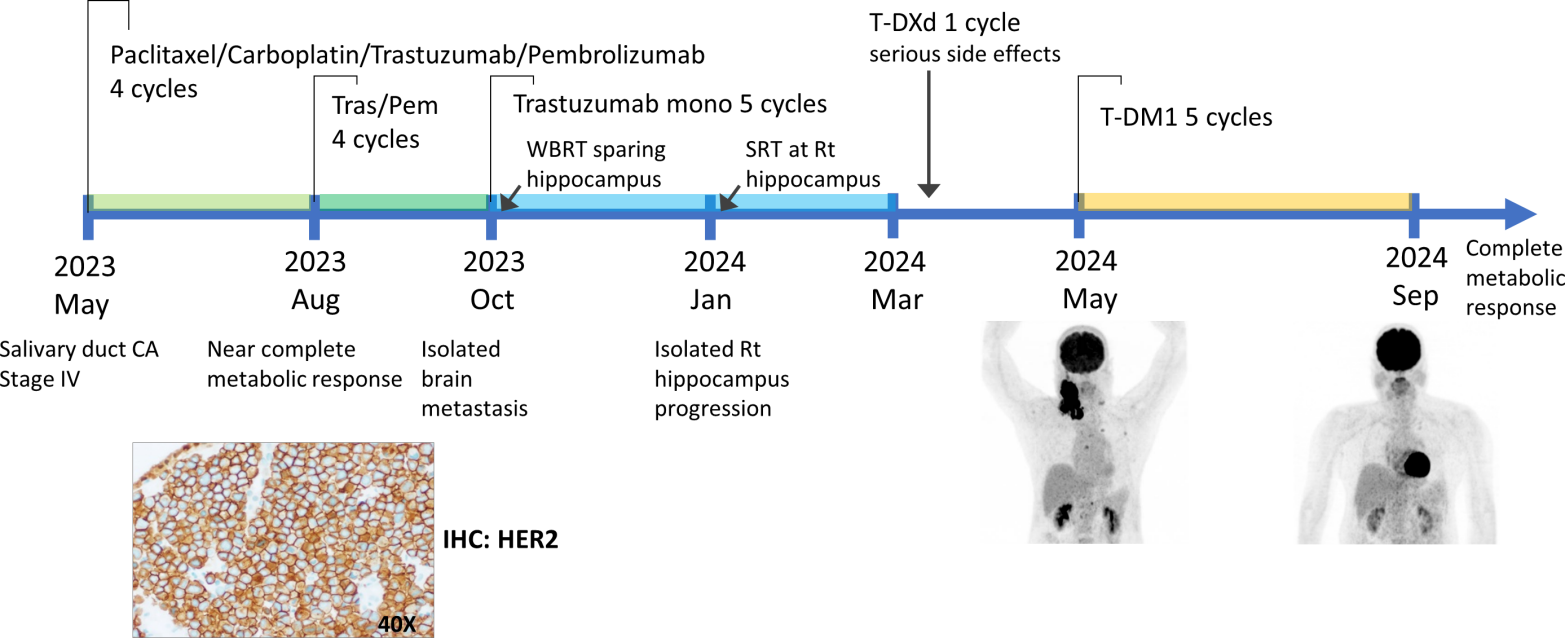
Timeline of diagnosis and treatment and HER2 IHC of case 2.

Case 2 A 65-year-old male diagnosed with metastatic salivary duct carcinoma, initially presented with multiple cervical lymphadenopathies. Imaging with PET-CT revealed a parotid gland mass along with widespread bone metastases. Histopathological analysis of the lymph node biopsies revealed a triple-positive profile characteristic of salivary duct carcinoma, including strong GATA3 expression, strong nuclear androgen receptor (AR) immunoreactivity. HER2 overexpression (IHC 3+ [performed on a metastatic specimen]) (**Figure 2**). HER2 status was determined by IHC using anti-HER2/neu (4B5, Ventana). HER2 expression was interpreted according to the 2016 ASCO/CAP gastric and gastroesophageal junction adenocarcinoma guidelines (8). NGS was performed using the Oncomine Comprehensive Assay Plus (a 517-gene CGP panel). Results revealed an *ERBB2* amplification (33.7 copies, no detectable mutation) and a high tumor mutational burden (TMB) of 10.55 mutations/Mb. The patient received systemic therapy with trastuzumab, pembrolizumab, paclitaxel, and carboplatin for four cycles, followed by maintenance with trastuzumab and pembrolizumab for eight additional cycles. This regimen achieved a complete metabolic response in all metastatic lymph nodes and bone lesions and a near-complete metabolic response at the primary parotid tumor. After 5 months of remission, the patient developed an isolated brain metastasis, for which whole-brain radiotherapy was administered alongside trastuzumab monotherapy, leading to a complete intracranial response.

However, three months later, new lesions emerged in the brain, left adrenal gland, and left iliac bone. He underwent stereotactic brain irradiation, followed by a single cycle of T-DXd. Unfortunately, treatment was complicated by grade ≥3 cytopenia, small bowel obstruction, and interstitial pneumonitis, suspected to be T-DXd related toxicities. Consequently, T-DXd was permanently discontinued. These toxicities created a concern for the patient and the care team. Subsequent treatment with T-DM1 for five cycles resulted in a complete response, as confirmed by PET-CT and brain Magnetic Resonance Imaging (MRI) (**Figure 2**). Following the physical hardships of his initial treatment, the switch to T-DM1 yielded a life-changing complete response. This breakthrough allowed him to return to the comfort of his home country, where he continues to reclaim his daily life with ongoing clinical benefit.

Case 3 A 28-year-old male presented with a large soft tissue mass on the back. His clinical course was acutely complicated by severe headache and emesis; brain MRI revealed oligometastatic disease with significant perilesional edema and secondary hydrocephalus. To alleviate mass effect and hydrocephalus, the patient underwent an emergency suboccipital craniotomy for resection of a left cerebellar lesion. The soft tissue mass on the back was also surgically resected, followed by stereotactic brain radiotherapy. Histopathological analysis and IHC from back tissue confirmed a metastatic poorly differentiated carcinoma, with tumor cells staining positive for AE1/AE3, CK5/6, and CK7. Further IHC characterization showed strong CK7 positivity and weak CK20 expression, while TTF1, P40, P63, P53, and adipophilin were negative. Similarly, the brain tumor was histopathologically diagnosed as metastatic poorly differentiated carcinoma, staining positive for AE1/AE3, CK5/6, CK7, and focal p40, while S100 and CD117 were negative. Comprehensive radiological staging with PET-CT revealed widespread metabolically active disease, including cervical and mediastinal lymphadenopathy, bilateral pulmonary nodules, a right parietal lobe lesion, and a bony metastasis at the T3 vertebra. Given the extensive metastatic burden and the absence of an identifiable primary site despite exhaustive evaluation, the patient was diagnosed with a carcinoma of unknown primary (CUP).

**Figure 3:**
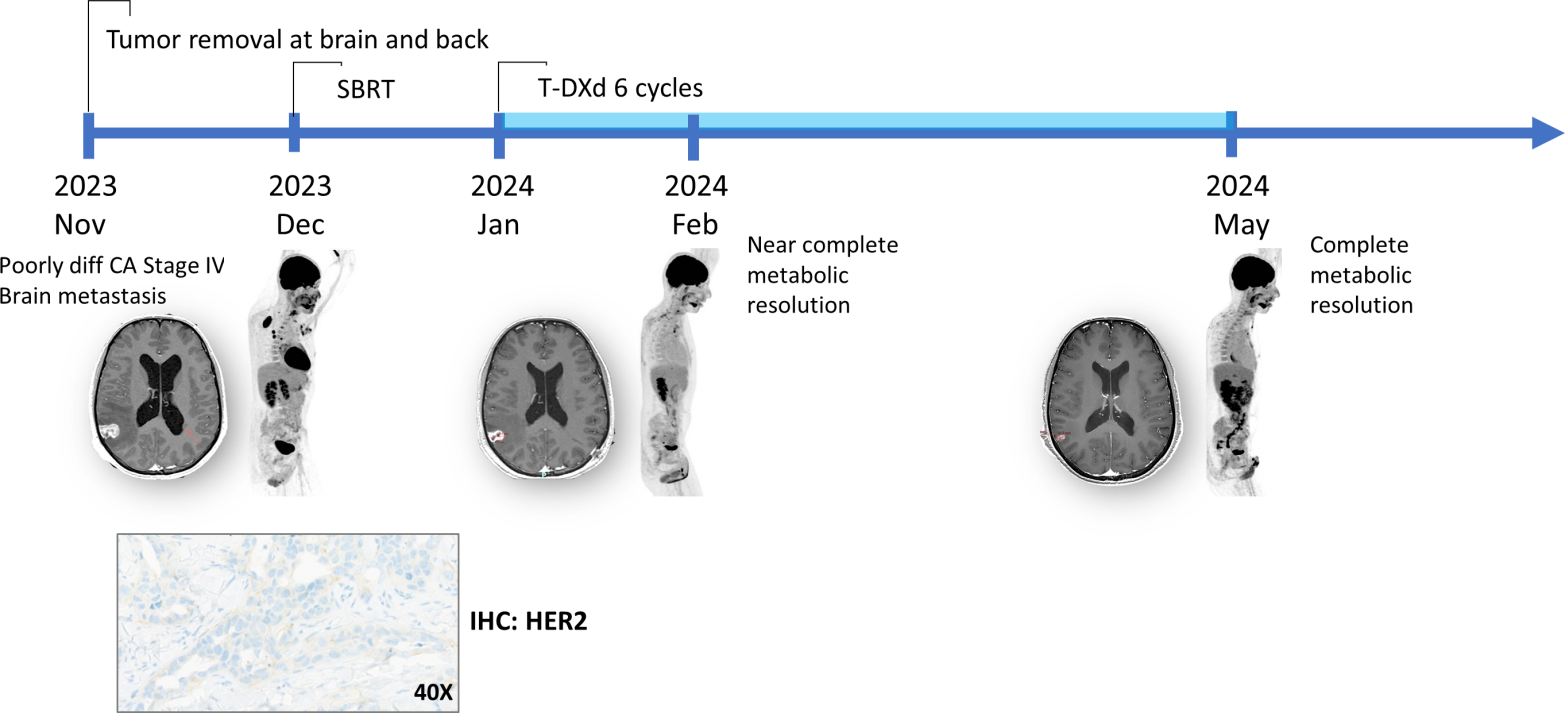
Timeline of diagnosis and treatment and HER2 IHC of case 3.

Molecular profiling *via* liquid biopsy-based NGS (Guardant360 CDx) identified *ERBB2* amplification (16.8 copies) and multiple *ERBB2* gene variants, including V1085L (3.9% variant allele frequency [VAF]), E695* (1.3% VAF), D796H (1.2% VAF), R677Q (0.5% VAF), T657I (0.3% VAF), and a *PIK3CA* H1047R (12.1% VAF) mutation was detected. Despite these genomic alterations, IHC of the biopsied lesion showed low HER2 expression (IHC 1+ [4B5, Ventana]) based on 2018 ASCO/CAP breast cancer criteria (7). This was characterized by faint or barely perceptible, incomplete membrane staining in >10% of tumor cells (**Figure 3**). Systemic therapy with T-DXd was initiated because of the high copy number of *ERBB2* amplification. Following the first cycle of T-DXd, the patient experienced grade 3 nausea and emesis, which led to secondary acute kidney injury (AKI) and fatigue. With the administration of antiemetic therapy and intravenous fluid support, the symptoms and renal function fully recovered. Subsequent cycles of T-DXd were well-tolerated with optimized supportive care. Following six cycles over a five-month period, the patient achieved a complete intracranial response and a near-complete response at the back site and other metastatic lesions (**Figure 3**). Aside from the initial grade 3 events, no further significant treatment-related adverse events were reported during subsequent cycles. The patient remains on ongoing T-DXd therapy with sustained clinical benefit in his home country.

## Discussion

Growing clinical evidence of the tumor-agnostic utility of HER2-targeted therapies encourages exploration of anti-HER2 agents—particularly trastuzumab deruxtecan (T-DXd)—in non-breast, non-gastric solid tumors. Multiple clinical trials, including DESTINY-PanTumor02 (1, 9), have demonstrated clinically meaningful response rates across diverse tumor types—biliary tract, bladder, cervical, endometrial, ovarian, pancreatic, and colorectal cancers. In these pan-tumor cohorts, objective response rates ranged from 26.5% to 37.1% among all patients and 51.4% to 61.3% in those with IHC 3+, with many patients achieving durable responses. These findings are transformative in precision oncology because they confirm that histology-agnostic treatment is viable when anchored in robust biomarker and therapeutic rationale. They also show benefit in patients whose HER2 expression falls below the traditional IHC 3+ threshold, challenging the binary positive/negative paradigm and underscoring the need for a continuum-based grading strategy to optimize patient selection.

While DESTINY-PanTumor02 focused on seven predefined cohorts of common and less-common cancers, our series expands the tumor-agnostic evidence to rare and orphan malignancies. Specifically, although the DESTINY-PanTumor02 biliary tract cohort included a small subset of patients with ampulla of Vater tumors, our study expands on this by highlighting a periampullary carcinoma case with an exceptionally high *ERBB2* copy number (77 copies). While Case 2 displayed a classic IHC 3+ profile similar to the high-responders in DESTINY-PanTumor02, the identification of a massive 33.7-copy amplification and high tumor mutational burden (TMB) underscores the high oncogenic dependency in this histology. Our finding of a near-complete response in CUP reinforces the feasibility of a molecularly driven, site-agnostic approach even when a primary tissue of origin is unidentified.

Our case series includes three patients with rare cancers—one with IHC 3+ and two with IHC < 3 +. All three tumors, however, harbored very high-level *ERBB2* amplification by NGS. Although NGS provides comprehensive genomic insights, its clinical utility for HER2-targeted therapy remains a subject of ongoing discussion due to frequent discordance with traditional IHC/ISH (reported in up to 10% of cases), a lack of universally standardized copy-number thresholds for defining positivity across diverse histologies, and limited prospective data validating NGS as a standalone companion diagnostic. Proposed NGS copy-number thresholds for *ERBB2* amplification vary widely, ranging from 3.2 to 6.0 copies depending on the specific assay and tumor type (10). However, the extreme *ERBB2* copy numbers identified in our series (16.8 to 77 copies) far exceed these controversial cut-offs. Such robust, high-level amplification likely represents a definitive genomic driver that transcends the limitations of unstandardized IHC scoring in rare tumors, thereby providing a reliable biomarker for ADC efficacy even in the absence of traditional protein overexpression.

No standardized guidelines currently exist for HER2 evaluation in rare cancers, and IHC panels are often unvalidated in these settings. Tissue scarcity and diagnostic urgency further complicate biomarker assessment. NGS overcomes these challenges by simultaneously detecting *ERBB2* amplification, activating *ERBB2* mutations, and co-occurring oncogenic alterations (e.g., *PIK3CA, TP53, CCNE1*), enabling comprehensive molecular profiling and timely, personalized treatment recommendations via molecular tumor boards. HER2-directed antibody-drug conjugates (ADCs) represent a promising therapeutic strategy for rare malignancies lacking established standards of care. *ERBB2* amplification identifies actionable subgroups across the rare types of cancer, where targeted payload delivery maximizes anti-tumor efficacy while minimizing systemic toxicity. Case 3 exemplifies the critical role of comprehensive molecular profiling in poorly differentiated carcinomas with ambiguous histology. In such orphan malignancies, traditional morphology and IHC often fail to identify a primary site or actionable targets. By shifting the diagnostic focus from the tissue of origin to targetable genomic drivers, NGS facilitates the use of potent therapies like T-DXd, significantly improving outcomes for patients with otherwise limited therapeutic options. Interestingly, in case 2, salivary duct carcinoma strikingly parallels HER2-positive breast cancer, providing a biological rationale for HER2-targeted therapy. Both malignancies share a distinct molecular signature: *ERBB2* amplification, AR expression, and a high frequency of *PIK3CA* mutations (11). Histologically, salivary duct carcinoma mirrors high-grade invasive ductal carcinoma of the breast, characterized by ductal architectures and central comedo-necrosis (12). This clinicopathological mimicry suggests the potential for translating established breast cancer treatment strategies to this rare salivary gland malignancy (13).

Our case series demonstrates that high-level *ERBB2* amplification (ranging from 16.8 to 77 copies) serves as a potent predictor of ADC efficacy, even when protein expression falls below the traditional thresholds established in major clinical trials. Unlike DESTINY-PanTumor02, which required IHC 3+ or 2+ for enrollment, our case 3 achieved a near-complete response despite an IHC 1+ (HER2-low) status, suggesting that a high gene copy number can override low phenotypic expression. Furthermore, while DESTINY-Lung01 (14) targeted *HER2*-mutated or overexpressing (IHC 2+/3+) cohorts and DESTINY-CRC02 (15) focused on IHC-positive colorectal cancer, our cohort achieved meaningful responses across rare histologies—including periampullary and salivary duct carcinomas—despite the presence of co-occurring mutations such as *PIK3CA* H1047R and *CCNE1* amplification. These results advocate for a genomic-first selection model, suggesting that quantitative NGS copy numbers may be more sensitive than IHC in identifying “hidden” responders in rare, driver-positive malignancies.

In addition, this series showcases the efficacy of HER2-targeted ADCs in the setting of biomarker discordance (IHC 1+/NGS+ [case 3]). The near-complete response in case 3, driven by high-level *ERBB2* amplification (16.8 copies) despite an IHC 1+ status, suggests that NGS may be a more sensitive predictor of clinical benefit in rare histologies where IHC scoring is unstandardized. Finally, our findings provide rare evidence for sequential ADC management; case 2 illustrates a successful clinical pivot to T-DM1 as salvage therapy following T-DXd-induced grade ≥3 interstitial pneumonitis. Furthermore, the complete intracranial responses observed in our salivary duct and poorly differentiated carcinoma cases add granular longitudinal data to the aggregated results reported in larger trials. Following the FDA’s recent tumor-agnostic approval of T-DXd, these results underscore the necessity of integrating NGS and Molecular Tumor Boards into diagnostic workflows. Ultimately, this series reinforces a precision-oncology model that prioritizes actionable genomic alterations over traditional tissue of origin, advocating for expanded HER2 testing to optimize outcomes in rare, driver-positive malignancies. This series also offers insights into the patient journey, a dimension often lacking from clinical trial reports.

Despite these promising observations, several limitations must be acknowledged. Our findings are based on a small, retrospective cohort of three patients, which restricts the generalizability of the results. Furthermore, the absence of standardized HER2 scoring systems for these specific rare histologies and the relatively short follow-up period necessitate caution. Prospective studies with larger cohorts are required to define the optimal *ERBB2* amplification thresholds and the long-term efficacy of sequential ADC strategies.

## Data Availability

The original contributions presented in the study are included in the article/supplementary material. Further inquiries can be directed to the corresponding author.
